# Improved renal cancer prognosis among users of drugs targeting renin-angiotensin system

**DOI:** 10.1007/s10552-021-01527-w

**Published:** 2021-12-18

**Authors:** Tommi Eskelinen, Thea Veitonmäki, Andres Kotsar, Teuvo L. J. Tammela, Antti Pöyhönen, Teemu J. Murtola

**Affiliations:** 1 Faculty of Medicine and Health Technology, Tampere University, Tampere, Finland; 2 Department of Urology, TAYS Cancer Center, Tampere, Finland; 3 Department of Urology, Tartu University Hospital, Tartu, Estonia; 4 Centre for Military Medicine, The Finnish Defence Forces, Helsinki, Finland

**Keywords:** Epidemiology, Pharmaco-epidemiology, Renal cell cancer, Prognosis

## Abstract

**Purpose:**

We explored renal cell cancer (RCC) survival among users of antihypertensive medication as hypertension is proposed to be a risk factor for RCC and ACE-inhibitors and angiotensin receptor blockers (ARBs) have been associated with improved prognosis of RCC.

**Methods:**

Finnish cohort of 13,873 participants with RCC diagnosed between 1995–2012 was formed from three national databases. RCC cases were identified from Finnish Cancer Registry, medication usage from national prescription database and co-morbidities from Care Registry of Healthcare. Logistic regression was used to calculate odds ratios for metastatic tumor extent at the time of diagnosis. Risk of RCC specific death after diagnosis was analyzed using Cox regression adjusted for tumor clinical characteristics.

**Results:**

A total of 5,179 participants died of RCC during the follow-up. No risk association was found for metastatic tumor extent for any drug group. ACE-inhibitors, but no other drug group were associated with decreased risk of RCC specific death overall (HR 0.88, 95% CI 0.82–0.95) compared to non-users. In time-dependent analysis high-dose use of ACE-inhibitors (392 Defined Daily Dose (DDD)/year), HR 0.54, 95% CI 0.45–0.66) and ARBs (786.1 DDD/year, HR 0.66, 95% CI 0.50–0.87) associated with improved RCC survival. No information of TNM-classification or tobacco smoking was available.

**Conclusion:**

ACE-inhibitors and ARBs in high dose associated with improved RCC specific survival. This may reflect overall benefit of treating hypertension with medication targeting renin-angiotensin system (RAS) system among RCC patients. Further studies are needed to explore the role of RAS in RCC.

**Supplementary Information:**

The online version contains supplementary material available at 10.1007/s10552-021-01527-w.

## Introduction

Incidence of renal cell cancer (RCC) has rather constantly increased since year 2000 worldwide. Slight decrease of incidence has been observed among women but increased incidence among men. [1, 2] A total of 963 cases in 2015 makes RCC the 9th most common cancer among men and 14th among women in Finland. Worldwide a total of 400,000 cancer cases were reported in 2018. However, only a few risk factors for RCC are known, male gender being one. Smoking and hypertension are also assumed to be RCC risk factors. [3, 4] Hypertension has been associated both with increased risk of RCC diagnosis and with worse disease-specific survival. [5] Role of antihypertensive medication as a RCC risk factor, however, is unknown.

A population-based study of Finnish citizens charting health behavior and general health reported that among middle-aged people over 50% in both genders have either diagnosed hypertension or are using antihypertensive drugs, with the proportion increasing among elderly citizens [6]. Seven different active substances of antihypertensive medications are listed in the most sold medication in Finland 2016, while the purchases have steadily increased.

Use of antihypertensive medication has been associated with the increased risk of developing RCC, although the topic is controversial and it is proposed to some extent reflect the risk association between hypertension and RCC [7, 8].

Recent study results propose hypertension to be an independent risk factor for the development of RCC. A long-term Norwegian study reported that the risk of RCC increases together with the elevation of blood pressure, increasing the risk up to twofold [9]. The observation was coherent with larger European cohort study, in which elevated blood pressure was found to be an independent risk factor even after smoking and antihypertensive medication use were considered [10]*.* A case–control study conducted in Canada found that especially the use of angiotensin receptor blockers (ARB) and ACE-inhibitors were associated with increased risk of kidney cancer especially after three years of exposure [11].

The mechanism of action varies between active substances and drug groups. In previous studies mechanisms of actions have been presented in molecular basis [12]. Especially ARBs and ACE-inhibitors are highlighted as most beneficial groups, but knowledge of their independent impact on tumor progress is limited since mostly they are combined with some other antihypertensive medication drug group. [12]

We performed a nationwide cohort study in order to clarify the population-based risk association between antihypertensive medication and renal cell cancer prognosis while considering simultaneous use of multiple drug groups with differing mechanisms of action. We focused our interest in two main outcomes – tumor extent at the time of diagnosis and the risk of RCC-specific death.

## Materials and methods

Study cohort consisting of 13,873 patients was obtained from the national Finnish Cancer Registry [13]. Each cohort participant was diagnosed with renal cell cancer in Finland between 1995–2012. Information on participants included personal identification number (ID), gender, date of birth and diagnosis together with possible date of death (Supplementary table 1). Information of cancer included detection method, tumor lateralization, primary treatment, and tumor morphological characteristics (clear cell carcinoma, chromophobe carcinoma or not known). Tumor extent was recorded either as localized disease with possible local nodular growth or distantly metastatic disease. Only malignant tumors were included. Cause of death was recorded according to ICD-10-classification (ICD-9 for 1995). ICD-10 code C64 recorded as the primary cause of death was classified as RCC-specific death.

The Social Insurance Institute of Finland (SII) registers each physician-prescribed medication purchases in Finland to prescription database. The database includes ATC-code of the purchased active substance, number of purchased packages and strength (mg amount) of doses for each separate purchase. Information of medication purchases was available for 1995–2012. Yearly total amount of purchases of each drug was calculated for each cohort participant by adding together all purchases within a given calendar year. This procedure allowed us to define exact amount of medication use before and after the year of RCC diagnosis. Cholesterol-lowering drug use and antidiabetic medication use were also obtained from the SII database as surrogates for diabetes and dyslipidemia.

The SII database information was applied to calculate the yearly cumulative amount of medication use during the follow-up. Cumulative mg amount for each calendar year was calculated separately for each antihypertensive drug and standardized by dividing with the drug-specific Defined Daily Dose (DDD) to obtain yearly number of doses. [14] Doses from drugs with similar mechanism of action were combined; β-blockers, ACE-inhibitors, angiotensin receptor inhibitors (ARBs), diuretics and calcium channel blockers (CCB).

Each cohort participant was regarded as a non-user until the first medication purchase. After the first purchase participant remained in the user-category until the end of the follow-up; either death, migration from Finland or the end of the year 2012, whichever came first. The status remained as such even if purchases ceased in order to minimize bias due to selective discontinuation of medication use at palliative phase of cancer treatment.

Each year with recorded medication purchases was regarded as a year of usage independent of the amount purchased. During the follow-up yearly DDD amount and number of years of usage were added together for total cumulative DDDs and years of use, respectively. Yearly dosing was evaluated by calculating intensity of usage by dividing yearly cumulative DDDs with cumulative number of years of usage. Intensity was stratified into three tertiles.

We applied Charlson Comorbidity Index (CCI) to evaluate overall 10-year prognosis of our cohort participants based on comorbidities. [15] CCI consists of different disease conditions and summarizes overall health status and offers percentage of likelihood for the10-year survival.

Logistic regression model was used to compare the risk between users of anti-hypertensive medication and non-users for being diagnosed with metastatic RCC. The model was adjusted for gender, age at baseline and further adjusted for comorbidities (diabetes and dyslipidemia). In this analysis antihypertensive medication use before RCC diagnosis was used as dichotomous variable; any use vs no use. Participants with missing information on tumor extent were excluded from this analysis.

To analyse the risk of RCC-specific death Cox regression was applied and adjusted for age at diagnosis, gender, comorbidities, tumor extent at diagnosis and primary RCC treatment. Treatment method was categorized as curative-intent surgery, palliative surgery, radiation therapy and other methods as the primary treatment. Time metric was years and months of follow-up since RCC diagnosis. The main outcome variable was RCC death during the follow-up.

Antihypertensive medication use was analyzed as time-dependent variable. Each drug group formed a separate time-dependent variable to model simultaneous use. Medication usage status as well as the DDD amount, years and intensity of medication use were prospectively updated annually according to the recorded medication purchases. The status of medication use was analyzed as a dichotomous variable. Additionally, to estimate dose-dependent risk trends the medication users were stratified by tertiles of cumulative DDD amount, years of use and intensity of use. Non-users were the reference group in all analyses. Using time-dependent variables allowed us to control for immortal time bias.

We performed analysis on cohort participants diagnosed before or after 2007 separately, to clarify the risk association of antihypertensive medication and the RCC prognosis since the prognosis may vary because of the current targeted therapies. Most of the current targeted therapies were combined into medical therapy after that year and became reimbursed by SII after 2007, so we chose that year as a cutline. Use of TKI-medication (tyrosine kinase inhibitor) and mTOR-inhibitors (mammalian target of rapamycin) was obtained from SII database. Additionally, we analyzed whether antihypertensive medication associates with decreased risk of at the later stage of diseases. We included only localized RCC on that analysis and time-metric was years into first purchase of TKI-medication or mTOR-medication.

All statistical tests were conducted using IBM-SPSS 25 (Statistical Package for Social Sciences) statistical software. [16] All reported p-values are two-sided.

## Results

### Population characteristics

Our cohort consisted of 7,720 men and 6,153 women. (Table 1) 10,953 participants had purchased antihypertensive medication at least once between years 1995–2012. There was no statistical difference between men and women considering antihypertensive medication use. The mean follow-up period was 6.19 years after RCC diagnosis. Mean Charlson Comorbidity index for cohort participants with or without antihypertensive medication was 4 (*p* < 0.05).

**Table 1:** Study cohort characteristics of patients diagnosed with RCC in Finland between 1995–2012

	Total, n	User of antihypertensive medication	Non-user of antihypertensive medication
Cohort	13,873	10,953 (79.0%)	2,920 (21.0%)
Men	7,720 (55.6%)	5,899 (76.4%)	1,821 (23.6%)
Women	6,153 (44.4%)	5,054 (82,1)	1,099 (17.9%)
Age at diagnosis, mean	67.2	69.1	60.2
Deaths overall	8,541 (61.6%)	6,753	1,788
RCC as cause of death	5,179 (37.3%)	3,921	1,258
Overall years of follow-up, median	6.20	6.12	6.49
Comorbidities			
Dyslipidemia	5,033	4,705(43.0%)	328 (11.2%)
Diabetes	3,123	2,912 (26.6%)	211 (7.2%)
Obesity	265	247 (2,3%)	18 (0.6%)
RCC stage at diagnosis			
Local/local nodular	6,341 (45.7%)	5,117 (46.7%)	1,224 (41.9%)
Distantly metastatic	4,588 (33.1%)	3,516 (32.1%)	1,072 (36.7%)
Unknown	2,944 (21.2%)	2,320 (21.2%)	624 (21.4%)
Primary treatment			
Surgery	6,624 (47.7%)	5,375 (49.1%)	1,249 (42.8%)
Palliative surgery	1,351 (9.7%)	996 (9.1%)	355 (12.2%)
Cytostatic/radiation	934 (6.7%)	676 (6.2%)	258 (8.8%)
Other	154 (1.1%)	129 (1.2%)	25 (0.9%)
Unknown	4,810 (34.7%)	3,777 (34.5%)	1,033 (35.4%)

### Tumor extent at diagnosis

Decreased risk of metastatic tumor extent at diagnosis was observed among users of calcium channel blockers in age-adjusted logistic regression (OR 0,87, 95% CI 0.77–0.98) (Table 2). There was no statistical significance in any other drug group. None of the drug groups remained associated with advanced RCC extent at diagnosis in multivariable adjusted model.

**Table 2:** The risk of distantly metastatic RCC at the time of diagnosis

	OR (95% CI) age-adjusted	OR (95% CI) multivariable-adjusted
ACE-inhibitors	0.93 (0.81–1.06)	1.01 (0.88–1.16)
AR-blockers	1.78 (0.90–3.50)	1.87 (0.94–3.72)
Ca2-channel blockers	0.87 (0.77–0.98)*	0.92 (0.81–1.05)
Diuretics	0.99 (0.88–1.12)	0.95 (0.84–1.08)
β-blockers	0.92 (0.83–1.03)	1.02 (0.91–1.14)

A cohort of 13,873 newly diagnosed patients with RCC in Finland between 1995–2012

Values reaching statistical significance (*p* < 0.05) are marked*

### Risk of RCC death

In age-adjusted analysis use of diuretics was associated with increased risk of RCC death (HR 1.10, 95% CI 1.01–1.19) (Table 3). After further adjustment for comorbidities, tumor extent and primary treatment the risk association persisted for diuretics (HR 1.10, 95% CI 1.01–1.19) and ACE-inhibitors (HR 1.10, 95% CI 1.01–1.21). No significant risk differences were observed for other drug groups in multivariable-adjusted model.

**Table 3:** The overall risk of RCC specific death and different antihypertensive medication

	HR (95% CI)age-adjusted	HR (95% CI)multivariable-adjusted
ACE-inhibitors	1.04 (0.95–1.14)	1.10 (1.01–1.21)*
ARB	0.91 (0.53–1.58)	0.86 (0.50–1.49)
Ca2-channel blockers	0.92 (0.85–1.01)	1.00 (0.91–1.09)
Diuretics	1.10 (1.01–1.19)*	1.10 (1.01–1.19)*
B-blockers	0.99 (0.92–1.06)	1.06 (0.98–1.14)

A cohort of 13,873 newly diagnosed patients with RCC in Finland between 1995–2012

HRs with statistical significance (*p* < 0.05) are marked with*

In time-dependent Cox regression the risk association with overall use of antihypertensive medication after RCC diagnosis was ambivalent. Consistently the association was protective in age- and gender-adjusted analysis (Table 4). Only exception was the use of diuretics (HR 1.13, 95% CI 1.06–1.20). Further adjustment for comorbidities, tumor extent and primary treatment altered the risk association, since the risk association of diuretics did not persist. Instead, use of β-blockers associated with increased risk of RCC death (HR 1.08, 95% CI 1.01–1.14), whereas CCBs and ACE-inhibitors were associated with decreased risk (HR 0.78, 95% CI 0.72–0.83; HR 0.88, 95% CI 0.82–0.95, respectively). A borderline significant risk decrease was observed also among ARB users (HR 0.91, 95% CI 0.83–1.00).

**Table 4:** The overall hazard ratio (HR) between RCC specific death and antihypertensive medication in time-dependent analysis. Cohort of 13,873 newly diagnosed patients with RCC in Finland between 1995–2012

	HR (95% CI)age-adjusted	HR (95% CI)multivariable-adjusted
ACE-inhibitor	0.76 (0.71–0.82)	0.88 (0.82–0.95)
Angiotensin receptor blocker	0.69 (0.63–0.76)	0.91 (0.83–1.00)
Calcium channel blocker	0.68 (0.63–0.73)	0.78 (0.72–0.83)
β-blocker	0.91 (0.85–0.97)	1.08 (1.01–1.14)
Diuretics	1.13 (1.06–1.20)	1.03 (0.97–1.10)

In analysis stratified by intensity of medication use, high-intensity (top tertile) use of ACE-inhibitors (392 DDD/year; HR 0.54, 95% CI 0.45–66) and ARBs (786.1 DDD/year; HR 0.66, 95% CI 0.50–0.87) was associated with decreased risk of RCC death in both age-adjusted and multivariable adjusted analysis (Table 5). Nevertheless, also high-intensity use of β-blockers and CCBs were associated with lowered risk of RCC death.

**Table 5:** Risk of RCC specific death and antihypertensive medication use stratified by intensity of use after RCC diagnosis. A cohort of 13,873 newly diagnosed patients with RCC in Finland between 1995–2012

	HR (95% CI)age adjusted	HR (95% CI)multivariable adjusted
*ACE-inhibitors, DDD amount*		
< 76.4	0.73 (0.67–0.79)	0.80 (0.74–0.87)
76.4–392.0	0.64 (0.58–0.73)	0.78 (0.69–0.88)
> 392.0	0.45 (0.37–0.54)	0.54 (0.45–0.66)
*ARB, DDD amount*		
< 205.3	0.60 (0.54–0.67)	0.75 (0.68–0.84)
205.3–786.1	0.67 (0.57–0.82)	0.86 (0.71–1.03)
> 786.1	0.46 (0.35–0.61)	0.66 (0.50–0.87)
*Ca2-channel blockers, DDD amount*		
< 61.3	0.71 (0.64–0.78)	0.76 (0.69–0.84)
61.3–331.0	0.65 (0.56–0.74)	0.75 (0.65–0.86)
> 331.0	0.85 (0.68–1.05)	1.12 (0.90–1.40)
*β-blockers*		
< 76.0	0.81 (0.76–0.87)	0.99 (0.92–1.06)
76.0–331.0	0.77 (0.69–0.85)	1.00 (0.90–1.12)
> 331.0	0.65 (0.56–0.77)	0.90 (0.77–1.06)
*Diuretics*		
< 124.5	1.06 (0.98–1.13)	1.06 (0.99–1.14)
124.5–568.2	0.97 (0.88–1.08)	1.08 (0.97–1.19)
> 568.2	0.94 (0.81–1.09)	1.03 (0.88–1.20)

These risk decreasing associations did not persist in lag time analyses as the risk decrease was not observed in ARBs or diuretics after one year of time lag (Table 6). Additionally, the risk decrease among ACE-inhibitor users was reversed to increased risk which persisted for up to five years’ time lag. A similar reversal of protective risk association was observed also for CCBs.

**Table 6:** Lag-time analysis of separate drug groups. Cohort of 13,873 newly diagnosed RCC patients in Finland between 1995–2012

	1-year lag	3-year lag	5-year lag
ACE-inhibitors	1.16 (1.08–1.24)	1.19 (1.10–1.27)	1.15 (1.07–1.24)
Angiotensin receptor blockers	1.17 (1.06–1.28)	1.17 (1.06–1.30)	1.08 (0.96–1.22)
Calcium channel blockers	0.98 (0.91–1.05)	0.98 (0.91–1.05)	0.99 (0.92–1.06)
β-blockers	1.12 (1.05–1.20)	1.12 (1.05–1.19)	1.10 (1.03–1.18)
Diuretics	1.11 (1.04–1.19)	0.98 (0.91–1.05)	1.05 (0.98–1.12)

### Subgroup analyses

The observed decreased risk association of ARBs and ACE-inhibitors persisted in subgroup analyses among men and women. Younger age was associated with improved prognosis among previously mentioned medication users. Even in distantly metastatic disease ACE-inhibitors associated with decreased mortality (HR 0.83, 95% CI 0.76–0.91). CCBs associated with improved prognosis (HR 0.78, 95% CI 0.68–0.91) together with ARBs and ACE-inhibitors when primary treatment was radical, curative intent surgery. See Table 7.

**Table 7:** Subgroup analyses in current cohort stratified by different drug groups of antihypertensive medication. Cohort of 13,873 RCC patients diagnosed in Finland between 1995–2012

HR (95% CI) multivariable-adjusted					
	ACE-inhibitors	ARB	CCB	β-blockers	Diuretics
*Gender*					
Male	0.73 (0.66–0.80)	0.69 (0.60–0.78)	0.80 (0.72–0.90)	0.93 (0.85–1.02)	1.11 (1.01–1.21)
Female	0.78 (0.70–0.87)	0.84 (0.74–0.96)	0.74 (0.66–0.84)	1.00 (0.91–1.11)	0.98 (0.89–1.08)
*Age at diagnosis*					
Under 69 years	0.66 (0.59–0.74)	0.65 (0.56–0.74)	0.83 (0.73–0.95)	0.95 (0.86–1.06)	1.11 (1.00–1.23)
Over 69 years	0.86 (0.78–0.94)	0.92 (0.81–1.04)	0.78 (0.70–0.87)	1.00 (0.92–1.09)	0.96 (0.87–1.04)
*Tumor extent at diagnosis*					
Local/local with nodular metastases	0.66 (0.57–0.77)	0.51 (0.42–0.61)	0.87 (0.74–1.03)	0.90 (0.77–1.05)	1.09 (0.93–1.27)
Distantly metastatic	0.83 (0.76–0.91)	0.99 (0.88–1.11)	0.83 (0.75–0.92)	1.10 (1.02–1.20)	1.01 (0.93–1.10)
*Tumor morphology*					
Clear cell carcinoma	0.69 (0.64–0.76)	0.68 (0.61–0.76)	0.82 (0.74–0.90)	0.99 (0.91–1.07)	1.10 (1.01–1.19)
Chromophobic	0.55 (0.08–3.33)	n/a	n/a	0.86 (0.05–13.43)	4.15 (0.29–59.45)
*Primary treatment*					
Radical surgery	0.65 (0.57–0.75)	0.56 (0.48–0.67)	0.78 (0.68–0.91)	0.93 (0.81–1.07)	1.05 (0.92–1.21)
Palliative surgery	0.77 (0.65–0.90)	1.02 (0.83–1.26)	0.88 (0.73–1.06)	0.99 (0.85–1.14)	0.94 (0.81–1.09)
Cytostatic/radiation	0.84 (0.70–1.02)	1.03 (0.81–1.30)	0.93 (0.75–1.15)	1.11 (0.93–1.32)	1.10 (0.92–1.31)

*CCB*: calcium channel blocker; *ARB*: angiontensin receptor blocker

Use of antihypertensive medication did not associate with decreased risk of metastases at the later stage. This was observed in each group of active substance. Use of ACE-inhibitors and ARBs was associated with decreased risk of RCC regardless of the diagnosis being after or before 2007. (Supplementary table 2).

## Discussion

Hypertension is a risk factor for development of renal cell carcinoma, although the background mechanism remains unclear. Antihypertensive medication is presumed to have prognostic value despite the mechanism being uncertain.

Our study shows a varying association between the use of antihypertensive medication and the risk of RCC-specific death. The risk was increased among users of diuretics while the use of ACE-inhibitors and ARBs seemed to decrease the risk. However, no long-term protective association was observed in lag time analyses. There was no association with tumor extent neither by any specific drug group nor by antihypertensive medication overall. High-dose use of multiple drug groups after RCC diagnosis was associated with lowered risk of RCC death supporting prognostic effect of intensive hypertension management.

Study reports are sparse if any considering association between antihypertensive medication and RCC extent at diagnosis and furthermore with prognosis. For other cancer sites, for example colorectal cancer (CRC), studies have reported tumor size to be reduced and tumors to be less often metastatic at diagnosis among ACE-inhibitor users [17]. Angiogenesis-dependent tumor growth and potential metastatic characteristic was found to be reduced when applying ACE-inhibitors in murine model [18]. Concordantly, a registry-based study found correlation between prescribed ACE-inhibitor use and reduced risk of distantly metastatic CRC, although the risk reduction could have been caused by more intensive screening [19]. Our study cohort demonstrated no risk reduction for metastatic RCC at diagnosis by any antihypertensive drug group. Therefore, the previous findings are unlikely to be generalizable from CRC to RCC and the potential benefits of renin-angiotensin system inhibition in RCC remains unclear.

Surgery remains the golden standard treatment for RCC in localized tumors. However, medical therapies used in management of advanced disease are developing rapidly and especially VEGF-targeted therapies develop hypertension as a common side-effect [20, 21]. It is thus presumable that the risk increase observed for several antihypertensive drug groups in lag-time analysis may reflect management of the side-effects in high-risk disease. On the other hand, antihypertensive drugs may interact with targeted RCC therapy. In previous studies patients who received VEGF-targeted therapy were reported to have improved cancer-specific survival when targeted therapy was combined with ACE-inhibitors or other RAS targeting active substance [20]. In our study cohort participants with high amount use of either ACE-inhibitors or ARBs had improved survival compared to non-users, supporting possible additive effect with RCC treatment. However, most participants with antihypertensive medication use had used ACE-inhibitors. Therefore, decreased risk association may also reflect the benefit of blood pressure management in general.

Our dataset includes only primary treatment, but distantly metastatic RCC cases accounted for a third of our study cohort. Thus, a notable portion of our cohort has likely received targeted therapy after primary treatment or palliative surgery [22, 23]. As effective VEGF treatment frequently induces hypertension, the inverse risk association with high-dose post-diagnostic medication use may indirectly reflect the effect of VEGF-therapy induced hypertension. On the other hand, in that case the risk decrease could be presumed to occur in high-dose use of any antihypertensive drug group. The risk decrease was observed only among users of ACE-inhibitors or ARBs supports the prognostic role of RAA-system inhibition specifically.

In previously mentioned Japanese study the use of either ACE-inhibitors or ARBs were associated with improved prognosis which is supported by our results. [18]. Additionally, our data demonstrates dose-dependency of the risk association, which was not reported in the previous study. The risk decrease was stronger in higher yearly doses and was observed among participants who used medication after RCC diagnosis. Such association was not observed with antihypertensive medication before RCC diagnosis. This suggests that treatment of hypertension with ACE-inhibitors or ARBs may have a role on survival by affecting disease progression at late stages, not on early stages of the disease progress.

Use of beta-blockers was not associated with RCC survival in a recent study of 913 beta-blocker users whose cancer was treated either with total or partial nephrectomy [24]. Concordantly, we observed that overall use of beta-blockers was not associated with elevated risk of RCC death. However, increased risk for RCC death was observed in high-dose use. Thus, the role of beta-blockers for RCC prognosis may depend on dosage. Worth noticing is differences between these two studies; our dataset included both metastatic and localized tumor extent, whereas in the previous study localized tumors were excluded.

Our results suggest beneficial association between renin-angiotensin system inhibiting medication and risk of RCC death. This benefit mainly concerns ACE-inhibitors and ARBs. The risk association is more favorable with more intensive use after RCC diagnosis. There was no risk association between tumor extent at diagnosis and medication use prior to RCC diagnosis. Dose-dependent risk association suggests causality. Risk of selection and immortal time bias need to be acknowledged. We conducted analysis of medication use as a time-dependent variable in which participants were categorized as non-users until first medication purchase to eliminate immortal time bias. To reduce the risk of selection bias due to selective discontinuation of antihypertensive drugs at terminal phase of cancer, exchanging back from user category to non-user category was not allowed.

Users of antihypertensive medication are in general likely to have more comorbidities and their general health status is more commonly unsatisfactory compared to non-users. Furthermore, the basic indication of prescribed antihypertensive medication depends on background comorbidities; diuretics as primary antihypertensive medication is more likely in people with concurrent heart disease compared to people with isolated hypertension without any heart conditions. This may have caused elevated mortality among antihypertensive medication, especially in diuretic users compared to non-users, but does not limit our inference of improved survival.

## Study strengths and limitations

The main strength of our study is a large nationwide cohort including both the localized and metastatic tumors. Specific information of drug purchases from the SII covering the identical time period as our cohort from Finnish Cancer Registry allowed us to estimate the risk associations definitively. However, our databases covered no information of RCC specific TNM-classification. We lacked information concerning possible adjuvant therapies following primary treatment. Cohort participant information did not include status on living habits such as tobacco smoking, physical activity or obesity. Their role in developing bias is possible and furthermore they may alter the risk association, likely elevating it.

## Conclusion

Our current findings demonstrate improved renal cell carcinoma specific survival among antihypertensive medication users after RCC diagnosis. The survival benefit is focused to users of ACE-inhibitors and ARBs, suggesting potential benefit of RAS inhibition. The risk decrease was not observed for usage occurring prior to RCC diagnosis. Our results support prognostic benefit of treating hypertension in RCC patients, especially drugs targeting RAS system may be beneficial. However, our study is retrospective, and further studies are needed to confirm our findings and to clarify the potential anticancer mechanism.

## Supplementary Information

Below is the link to the electronic supplementary material.

Supplementary file1 (DOCX 14 KB)

Supplementary file2 (DOCX 16 KB)
