# Supplementary material for: Improved renal cancer prognosis among users of drugs targeting renin-angiotensin system
Source: Cancer Causes Control. 2021 Dec 18;33(2):313–20. doi: 10.1007/s10552-021-01527-w (PMC8776666; doi:10.1007/s10552-021-01527-w)
Supplement: Supplementary file 1 — Supplementary file1 (DOCX 14 KB) [file 10552_2021_1527_MOESM1_ESM.docx]

| Personal information | - Gender - Personal identification number |
| --- | --- |
| Tumor extent (No full information of TNM-scale) | - Unknown - Local - Locally advanced (nodular) - Metastatic to other organs |
| Tumor morphology (ICD03-code) |  |
| Tumor primary location (ICD03-code) |  |
| Lateralization (kidney) | Left, right and/or both |
| Primary treatment | - Surgical - Palliative surgery - Radiation - Palliative radiation - Cytostatic - Hormone therapy - Other |
| Primary cause of death (ICD-10) | - Renal cell cancer |
|  | - Other |

**Supplementary table 1.** The information of renal cell cancer, tumor extent and treatment options received from Finnish Cancer Registry database. Cohort of patients diagnosed with RCC in Finland between 1995–2012.
