# Supplementary material for: Improved renal cancer prognosis among users of drugs targeting renin-angiotensin system
Source: Cancer Causes Control. 2021 Dec 18;33(2):313–20. doi: 10.1007/s10552-021-01527-w (PMC8776666; doi:10.1007/s10552-021-01527-w)
Supplement: Supplementary file 2 — Supplementary file2 (DOCX 16 KB) [file 10552_2021_1527_MOESM2_ESM.docx]

| **Patients diagnosed** | HR (95% CI) | **Localized RCC, follow-up to first purchase of mTOR** | HR (95% CI) |
| --- | --- | --- | --- |
| *Before 2007* |  | ACE-inhibitor | 1.09 (0.39–3.06) |
| ACE-inhibitors | 0.78 (0.71–0.85) | Diuretics | 0.82 (0.26–2.57) |
| Diuretics | 0.90 (0.83–0.97) | Angiotensin receptor blockers | 1.23 (0.32–4.76) |
| Angiotensin receptor blockers | 0.76 (0.66–0.87) | Beta-blockers | 0.51 (0.11–2.46) |
| Beta-blockers | 0.98 (0.90–1.06) | Calcium channel blockers | 3.85 (0.51–29.22) |
| Calcium channel blockers | 0.71 (0.65–0.77) | **Localized RCC, follow-up to first purchase of TKI** |  |
| *After 2007* |  | ACE-inhibitor | 0.88 (0.64–1.22) |
| ACE-inhibitors | 0.88 (0.78–0.99) | Diuretics | 1.04 (0.77–1.42) |
| Diuretics | 0.92 (0.83–1.03) | Angiotensin receptor blockers | 0.99 (0.70–1.40) |
| Angiotensin receptor blockers | 0.85 (0.75–0.97) | Beta-blockers | 0.90 (0.66–1.23) |
| Beta-blockers | 0.91 (0.82–1.02) | Calcium channel blockers | 0.81 (0.58–1.14) |
| Calcium channel blockers | 0.69 (0.62–0.77) |  |  |
| **Patients receiving targeted therapy** |  |  |  |
| mTOR-inhibitor | 1.12 (0.88–1.42) |  |  |
| Tyrosine kinasine inhibitor | 0.86 (0.60–1.24) |  |  |
|  |  |  |  |
|  |  |  |  |

**Supplementary table 2.** Subgroup analyses of conducted on participants receiving targeted therapy and risk association of antihypertensive medication on the risk of RCC death. Patients diagnosed with RCC before or after 2007, and their risk association of RCC death and antihypertensive medication. The risk of RCC death among participants with use of mTOR or TKI medication and antihypertensive medication. On the right paragraph the association between mTOR and TKIs and risk of metastatic RCC after diagnosis (initially only localized were selected) by different drug.
